# The Influence of Different Fever Definitions on the Rate of Fever in Neutropenia Diagnosed in Children with Cancer

**DOI:** 10.1371/journal.pone.0117528

**Published:** 2015-02-11

**Authors:** Roland A. Ammann, Oliver Teuffel, Philipp Agyeman, Nadine Amport, Kurt Leibundgut

**Affiliations:** 1 Department of Pediatrics, University of Bern, Bern, Switzerland; 2 Division of Oncology, Medical Services of the Statutory Health Insurance Baden-Württemberg, Tübingen, Germany; 3 Institute for Infectious Diseases, University of Bern, Bern, Switzerland; Mayo Clinic, UNITED STATES

## Abstract

**Background:**

The temperature limit defining fever (TLDF) is based on scarce evidence. This study aimed to determine the rate of fever in neutropenia (FN) episodes additionally diagnosed by lower versus standard TLDF.

**Methods:**

In a single center using a high TLDF (39.0°C tympanic temperature, Limit_Standard_), pediatric patients treated with chemotherapy for cancer were observed prospectively. Results of all temperature measurements and CBCs were recorded. The application of lower TLDFs (Limit_Low_; range, 37.5°C to 38.9°C) versus Limit_Standard_ was simulated *in silicon*, resulting in three types of FN: simultaneous FN, diagnosed at both limits within 1 hour; earlier FN, diagnosed >1hour earlier at Limit_Low_; and additional FN, not diagnosed at Limit_Standard_.

**Results:**

In 39 patients, 8896 temperature measurements and 1873 CBCs were recorded during 289 months of chemotherapy. Virtually applying Limit_Standard_ resulted in 34 FN diagnoses. The predefined relevantly (≥15%) increased FN rate was reached at Limit_Low_ 38.4°C, with total 44 FN, 23 simultaneous, 11 earlier, and 10 additional (Poisson rate ratio_Additional/Standard_, 0.29; 95% lower confidence bound, 0.16). Virtually applying 37.5°C as Limit_Low_ led to earlier FN diagnosis (median, 4.5 hours; 95% CI, 1.0 to 20.8), and to 53 additional FN diagnosed. In 51 (96%) of them, spontaneous defervescence without specific therapy was observed in reality.

**Conclusion:**

Lower TLDFs led to many additional FN diagnoses, implying overtreatment because spontaneous defervescence was observed in the vast majority. Lower TLDFs led as well to relevantly earlier diagnosis in a minority of FN episodes. The question if the high TLDF is not only efficacious but as well safe remains open.

## Introduction

Fever in severe chemotherapy-induced neutropenia (hereafter fever in neutropenia, FN), is the most frequent potentially lethal complication of therapy in patients with cancer [1]. Despite its clinical importance, however, fever and thus FN are not consistently defined in pediatric oncology. A widely used fever definition, specifically, temperature limits defining fever (TLDF), encompasses a persistent temperature ≥38.0°C, or a single temperature ≥38.3°C [2] or ≥38.5°C [3], but definitions used clinically and in research range from 37.5°C to 39.0°C [3–6]. This wide range reflects that both an international consensus on TLDF is missing [7], and that national consensus-based policies are incompletely implemented locally [3, 8].

**Figure 1:**
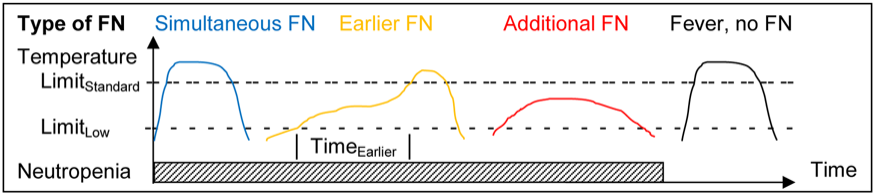
Types of FN diagnoses applying a low versus high TLDF.

The TLDF, however, directly influences whether FN is diagnosed or not, usually implying emergency hospitalization and intravenous broad-spectrum antimicrobial therapy as current standard of care [7]. The TLDF has thus important implications on individual patient management, health-related quality of life, resource utilization, costs, and potentially treatment-related mortality [9–11]. Efficacy must be weighed against safety for the determination of an ideal TLDF. A high TLDF emphasizes efficacy by avoiding unnecessary FN diagnoses in patients without relevant infections who will spontaneously defervesce (Fig. 1) [6]. A low TLDF emphasizes safety by avoiding delays in FN diagnosis and start of empirical antimicrobial therapy. Such delays may increase morbidity and mortality in patients with bacterial infection [12]. There is very scarce evidence how to rationally determine this ideal TLDF. To our knowledge, there is no published or ongoing prospective study on the efficacy or safety of different TLDFs in pediatric or adult oncology. A single Swiss two-center retrospective study reported no significant difference in the rate of FN, and of FN with bacteremia, between temperature limits of 38.5°C and 39.0°C [6].

Based on the historically established clinical use of a high TLDF of 39.0°C in Bern [6], this prospective single-center study aimed to assess the efficacy of a high TLDF by determining the rate of additional FN diagnoses when virtually lowering it. This aim was achieved.

## Patients and Methods

### Study Design

This was a prospective single-center observational study. The intervention, virtually lowering the TLDF, and its effects were simulated *in silicon*, i.e., using software on a personal computer, without changing the routine clinical management of patients. The study was conducted in accordance with the Declaration of Helsinki. Before starting patient accrual, the protocol was approved by the Institutional Review Board (Ethikkommission der Universitätskinderkliniken Bern) and registered at www.clinicaltrials.com (NCT01683370). Patients, if able to judge, and their legal guardians gave written informed consent prior to study entry.

### Patients

Patients aged 1 to 17 years with cancer who were treated at the Department of Pediatrics, University of Bern, Inselspital, Bern, Switzerland, and who required chemotherapy for ≥2 months at time of recruitment were eligible.

Patients were off study when informed consent was withdrawn, and when chemotherapy was completed (≥2 weeks after last dose, and absolute neutrophil count (ANC) >0.5 G/L).

### Routine Clinical Management, Including FN

Patients were treated with chemotherapy, including myeloablative therapy followed by autologous peripheral blood stem cell transplantation, or multimodal therapy, according to internationally established protocols.

Temperature was always measured in the ear by infrared tympanic thermometry using the Braun ThermoScan 5 (IRT 4520; Braun GmbH, Kronberg, Germany; steps displayed, 0.1°C; accuracy, ±0.2°C; clinical repeatability, ±0.14°C) [13, 14]. All parents were trained in its use during initial hospitalization and at study entry.

In inpatients, temperature was measured routinely twice a day. Additional measurements were made when fever was suspected, as well as before and during transfusions and medications known to potentially induce fever. In outpatients, parents were instructed to measure temperature when they suspected fever. If temperature was ≥39.0°C, or in case of reduced general condition or other problems, parents called the pediatric oncology department. An emergency complete blood count (CBC) was ordered if the last CBC was older than 48 hours. Patients with an ANC >0.5 G/L and in good general condition were allowed to take oral paracetamol for the next 48 hours. Those with an ANC ≤0.5 G/L or in reduced general condition were seen in the emergency department and usually hospitalized for FN.

FN was diagnosed when a patient had fever during a period of chemotherapy-induced severe neutropenia. Fever was defined as a single ear temperature ≥39.0°C (Limit_Standard_). With increasing or plateau temperature, this TLDF corresponds to 39.1°C core temperature, and to 38.4°C axillary temperature [13]. Severe neutropenia was defined as ANC ≤0.5 G/L, or ≤1.0 G/L and expected to decline. In reality, the treating physician was free to diagnose FN at lower temperatures if clinically indicated [2]. At diagnosis of FN, patients were hospitalized and treated with empirical intravenous broad-spectrum antibiotics, usually ceftriaxone plus amikacin, plus antipyretics. Further details of management have been published [6, 15].

### Study Specific Procedures

Participation in this observational study did not influence any diagnostic nor therapeutic decisions. The parents were instructed to note results of temperature measurements clinically indicated in the outpatient setting on paper forms.

An experienced pediatric oncology nurse (N.A.) extracted information from these forms and patient charts: time and results of temperature measurements and CBCs; number of emergency calls and CBCs performed for fever; FN diagnoses; and clinical course of FN. This information was checked for plausibility and agreement with charts by a pediatric oncologist (R.A.A.) before analysis.

### Definitions

The duration of antimicrobial therapy for FN defined the duration of FN episodes. Restarting antimicrobial therapy within 7 days and with persistent neutropenia was considered to belong to the same FN episode. The TLDF used in reality (Limit_Reality_) was defined as the first temperature ≥39.0°C, or the highest temperature measured until start of antibiotics if the FN diagnosis was made at lower temperatures. Adverse events (AE) were defined as published [15], and tracked onward for 7 days after end of FN.

For analysis, Limit_Standard_ was virtually replaced by a lower TLDF (Limit_Low_). Time_Earlier_ was defined as the difference in time of FN diagnosis applying Limit_Low_ versus Limit_Standard_. Three types of FN episodes were differentiated (Fig. 1). First, simultaneous FN was defined by Time_Earlier_ ≤1 hour. Second, earlier FN was defined by Time_Earlier_ >1 hour and ≤168 hours, with continued neutropenia and continued fever (temperature measured at least once ≥37.5°C every 24 hours) during Time_Earlier_. Third, additional FN was defined as FN diagnosed applying Limit_Low_, but not applying Limit_Standard_.

For analysis, the minimum delay between two emergency calls or CBCs for fever was assumed to be 48 hours. If virtual CBCs required by Limit_Low_ did not coincide with CBCs performed, virtual ANCs were calculated assuming linear changes over time, and that the ANC was 17% of the leukocyte count (previously unpublished data from reference 15).

### Statistics

Because of non-normally distributed data, median, interquartile range (IQR), and range were calculated. Fisher’s exact test, the exact Wilcoxon’s rank sum test, and Kaplan-Meier estimates with their 95% confidence interval (CI) were calculated where applicable [16]. Poisson rates with exact 95% CI were calculated. Rate ratios of additional and of earlier versus standard FN diagnoses (RR_Early/Std_, RR_Add/Std_) were calculated, together with their exact 95% lower confidence bound (LCB; lower bound of the exact 90% CI). Limit_Low_ was varied between 39.0°C and 37.5°C [17].

Because of missing evidence for the definition of time limits differentiating earlier versus additional, and earlier versus simultaneous FN, corresponding sensitivity analyses were performed. For the time limit differentiating earlier versus additional FN, sensitivity analyses used 72 hours and 999 hours instead of the 168 hours used for the main analysis. For the time limit differentiating earlier versus simultaneous FN, sensitivity analyses used 4 hours instead of 1 hour used in the main analysis. This resulted in a total of 5 (2*3–1) sensitivity analyses besides the main analysis.

In FN diagnosed in reality below Limit_Standard_, antipyretics preclude that temperature rises to Limit_Standard_ (Fig. 1), which would lead to an overestimation of additional FN episodes. This potential distortion was avoided by discarding information on temperature and CBC within 7 days preceding such episodes before main analysis. The clinical course of FN in reality, however, was described in the full dataset, i.e., without discarding information on these 7 days periods, in order to avoid artificial underreporting of antimicrobial therapy and of AE in FN defined by Limit_Low_, Sample size was determined in a power analysis by 1000-fold random simulation on data of 94 historical patients from Bern with 177 FN during 81.7 years chemotherapy exposure time [18]. Assuming a 33% increase of the FN rate by applying Limit_Low_ instead of Limit_Standard_, 32 FN episodes defined by Limit_Standard_ were found to reach 80% power to detect a clinically relevant increase of ≥15% in the FN rate (95% LCB of RR_Add/Std_ ≥0.15; α = 0.05).

The *in silicon* simulation by virtually applying different TLDFs was performed in Excel 2010 spreadsheets, and the statistical analyses in *R 2.15.1* (R Foundation for Statistical Computing, Vienna, Austria). *P*-values <0.05 were considered significant.

## Results

### Patients

This study was open for recruitment from August 2012 to May 2013. Of 40 eligible patients, 39 participated in the study. Their median age at recruitment was 7.4 years (range, 1.2 to 16.7), and 16 (41%) were girls. Diagnoses were acute lymphoblastic leukemia in 18 (46%) patients, acute myeloid leukemia in 2 (5%), Non-Hodgkin lymphoma in 2 (5%), central nervous system tumor in 5 (13%), and other solid tumors in 12 (31%). Four (10%) patients had relapses, and one (3%) a second malignancy.

### Chemotherapy, Temperature Measurements and CBC

The study was closed in August 2013 when the accrual goal of 32 FN episodes with fever ≥39.0°C (Limit_Reality_ ≥Limit_Standard_) was reached. The cumulative chemotherapy exposure time in 39 patients studied was 8799 days (289 months), with a median of 199 days per patient (range, 63 to 366).

During this time, temperature was recorded 8896 times, with a median rate of 26 measurements per patient per month (IQR, 8 to 53; range, 0 to 237). The median temperature measured was 37.1°C (IQR, 36.7 to 37.6; range, 35.0 to 41.2), and 283 (3.2%) temperatures were ≥39.0°C.

There were 1873 CBCs recorded, with a median rate of 6 CBC per patient per month (IQR, 4 to 8; range, 2 to 23). The ANC was ≤0.5 G/L in 435 CBCs (23%), 0.5 to 1.0 G/L in 244 (13%), >1.0 G/L in 1032 (55%), and unknown in 162 (9%).

### FN Episodes Diagnosed in Reality

In the 32 FN episodes diagnosed at temperatures ≥39.0°C, the ANC was ≤0.5 G/L in 28 (88%) episodes, and >0.5 G/L but ≤1.0 G/L and expected to decline in 4 (13%).

During the study, 11 further FN episodes, all at an ANC ≤0.5 G/L, were diagnosed at lower temperatures (range, 38.0°C to 38.9°C) for different clinical reasons (steroid therapy for ALL within 7 days before FN, 4; AML, 2; other, 5). Male sex was more frequent in the FN episodes diagnosed <39.0°C versus ≥39.0°C, while other characteristics of patients, disease, temperature measurements before FN diagnosis, and outcomes were not significantly different (Table 1).

**Table 1:** Characteristics and Outcome of 43 FN Episodes Diagnosed in Reality.

	**Temperature of FN diagnosis**		
**Characteristic / Outcome**	**≥39.0°C (N = 32)**	**<39.0°C (N = 11)**	***P***
Characteristics of patient and disease			
Age group			0.69
1.00 to 3.99 years	6 (19%)	2 (18%)	
4.00 to 7.99 years	13 (41%)	3 (27%)	
8.00 to 11.99 years	9 (28%)	3 (27%)	
≥12 years	4 (13%)	3 (27%)	
Male sex	14 (44%)	9 (82%)	0.039
Diagnosis			0.83
ALL, preceding corticosteroid therapy	10 (31%)	4 (36%)	
ALL, other preceding therapy	9 (28%)	2 (18%)	
Acute myeloid leukemia	3 (9%)	2 (18%)	
Other diagnoses than acute leukemia	10 (31%)	3 (27%)	
Temperature measurements before FN			
Number of TM within 24 hours	3 (1 to 6)	4 (2 to 8)	0.62
Time since last TM [hours]	1.0 (0.5 to 3.8)	1.4 (0.6 to 2.6)	0.99
Increase since last TM [°C per hour]	0.60 (0.20 to 1.01)	0.31 (0.15 to 0.45)	0.19
Outcomes			
Any adverse event	14 (44%)	5 (45%)	1.00
AE, Serious medical complication	1 (3%)	1 (9%)	0.45
AE, bacteremia	4 (13%)	4 (36%)	0.17
AE, any MDI	13 (41%)	5 (45%)	1.00
AE, pneumonia	4 (13%)	1 (9%)	1.00
Length of hospitalization [days]	5.7 (3.1 to 8.5)	8.2 (4.4 to 26.6)	0.18
Length of i.v. antibiotics [days]	5.5 (3.5 to 8.5)	7.2 (3.7 to 24.9)	0.38
Length of any antibiotics [days]	5.6 (3.7 to 8.7)	10.0 (4.5 to 29.0)	0.30

Indicated are numbers (proportion of all episodes) and *P*-values from Fisher’s exact test for categorical variables, and medians (interquartile range) and *P*-values from Wilcoxon’s rank sum test for continuously measured variables.

AE, adverse event; ALL, acute lymphoblastic leukemia; FN, fever in neutropenia; MDI, microbiologically defined infection; TM, temperature measurement.

In total, 43 (32+11) FN episodes were diagnosed in 20 of the 39 patients (median number per patient, 1; range, 0 to 5), at a rate of 0.15 per month of chemotherapy exposure time (95% CI, 0.11 to 0.20). The median Limit_Reality_ was 39.1°C (range, 38.0 to 40.2). In 35 episodes with temperature measurements recorded within 24 hours before FN diagnosis, the median calculated temperature increase preceding FN diagnosis was 0.40°C per hour (IQR, 0.16 to 0.96; range, 0.05 to 2.80).

Twice, FN was not diagnosed and intravenous empirical antimicrobial therapy was not initiated despite fever ≥39.0°C during neutropenia. Both patients were in ALL maintenance therapy, had been diagnosed with an upper airway infection with good general condition within 24 hours before fulfilling FN criteria, later received oral antibiotics, with uneventful clinical course.

### Emergency Calls and CBCs at Different TLDFs

In reality, 90 emergency calls for fever were recorded. Virtually applying different TLDFs, this number reduced to 65 (72%) at Limit_Standard_, and increased to 108 for 38.5°C (120%), to 161 for 38.0°C (179%), and to 360 for 37.5°C (400%). In reality, 59 emergency CBCs for fever were recorded. Virtually applying different TLDFs, this number reduced to 30 (51%) at Limit_Standard_, and increased to 55 for 38.5°C (93%), to 81 for 38.0°C (137%), and to 179 for 37.5°C (303%).

### Diagnoses of FN at Different TLDFs

Virtually applying different TLDFs, the number of episodes of fever (with or without neutropenia) increased from 124 at Limit_Standard_ to 291 (235%) at 38.5°C, to 604 (487%) at 38.0°C, and to 1191 (960%) at 37.5°C.

**Figure 2:**
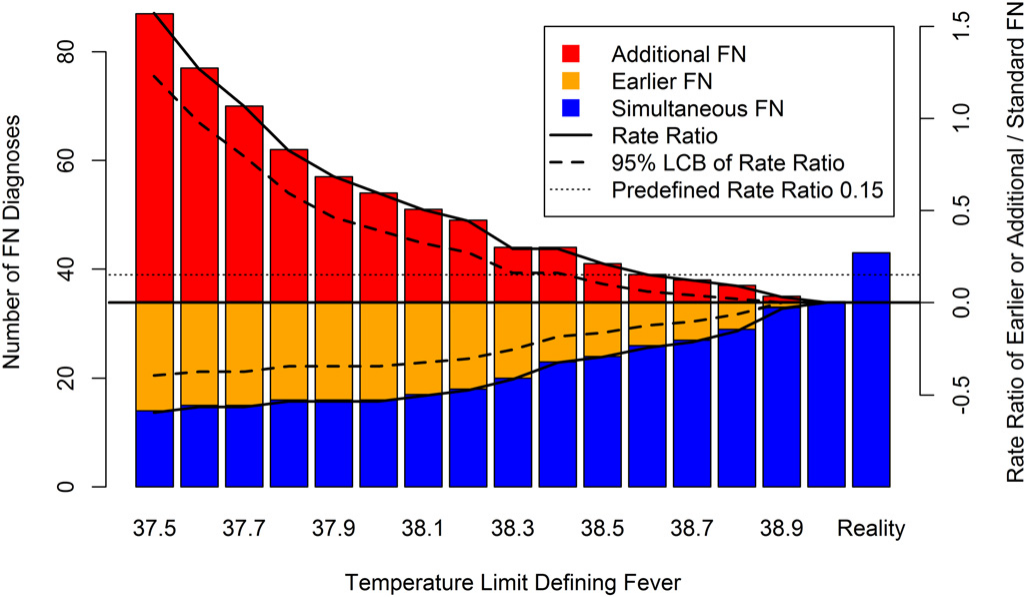
Additional, earlier, and simultaneous FN diagnoses according to TLDF.

The 7 days preceding the 11 FN episodes diagnosed at Limit_Reality_<Limit_Standard_ were now discarded from the dataset for the main analysis. Virtually applying Limit_Standard_, 34 (43–11+2) FN episodes remained. The number of FN episodes diagnosed by virtually applying Limit_Low_ increased to 41 at 38.5°C, to 54 at 38.0°C, and to 87 at 37.5°C (Table 2, Fig. 2).

**Table 2:** Frequency of FN Episodes Diagnosed in Reality, and Virtually at Different Temperature Limits Defining Fever.

	**FN diagnosed**	**FN virtually diagnosed using limit_Standard_ or limit_Low_**				
**Type of analysis, item**	**in reality**	**39.0°C**	**38.5°C**	**38.4°C**	**38.0°C**	**37.5°C**
Main analysis^a^ (time limits 168^b^, 1^c^)						
Total number of FN	43 (100%)	34 (100%)	41 (100%)	44 (100%)	54 (100%)	87 (100%)
Simultaneous FN	43 (100%)	34 (100%)	24 (59%)	23 (52%)	16 (30%)	14 (16%)
Earlier FN	0	0	10 (24%)	11 (25%)	18 (33%)	20 (23%)
Additional FN	0	0	7 (17%)	10 (23%)	20 (37%)	53 (61%)
Ratio additional / early FN	-	-	0.70	0.91	1.11	2.65
Rate ratio_Early/Std_ (95% LCB)	-	0.00	0.29 (0.16)	0.32 (0.18)	0.53 (0.34)	0.59 (0.39)
Rate ratio_Additional/Std_ (95% LCB)	-	0.00	0.21 (0.10)	0.29 (0.16)	0.59 (0.39)	1.56 (1.22)
Sensitivity analysis^a^ (time limits 999^b^, 1^c^)						
Ratio additional / early FN	-	-	0.70	0.82	0.88	1.37
Rate ratio_Early/Std_ (95% LCB)	-	0.00	0.32 (0.18)	0.35 (0.20)	0.55 (0.35)	0.61 (0.40)
Rate ratio_Additional/Std_ (95% LCB)	-	0.00	0.23 (0.11)	0.29 (0.15)	0.48 (0.30)	0.84 (0.59)
Sensitivity analysis^a^ (time limits 72^b^, 4^c^)						
Ratio additional / early FN	-	-	1.29	1.50	1.85	4.73
Rate ratio_Early/Std_ (95% LCB)	-	0.00	0.20 (0.09)	0.23 (0.11)	0.37 (0.22)	0.43 (0.26)
Rate ratio_Additional/Std_ (95% LCB)	-	0.00	0.26 (0.10)	0.34 (0.16)	0.69 (0.39)	2.03 (1.22)

FN, fever in neutropenia; LCB, lower confidence bound; RR_Add/Std_, rate ratio additional/standard; RR_Early/Std_, rate ratio earlier/standard.

^a^7 days preceding 11 FN episodes diagnosed at Limit_Reality_<Limit_Standard_ discarded from dataset;

^b^Time limit, in hours, differentiating earlier versus additional FN;

^c^Time limit, in hours, differentiating earlier versus simultaneous FN.

The predefined clinically relevant increase of ≥15% in the FN rate was reached when Limit_Low_ was 38.4°C, with 44 FN, 10 additional FN (RR_Add/Std_, 0.29; 95% LCB, 0.16), 23 simultaneous, and 11 earlier FN (Table 2, Fig. 2).

**Figure 3:**
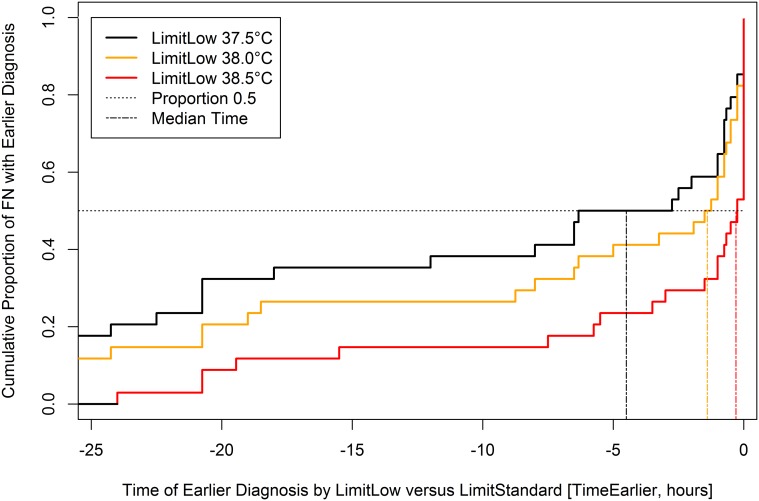
Cumulative proportion plots of Time_Earlier_ at different Limit_Low_ in 34 FN episodes diagnosed at Limit_Standard_.

In the 34 FN episodes defined by Limit_Standard_ the median Time_Earlier_ was 0.3 hours (95% CI, 0.0 to 1.5) at Limit_Low_ 38.5°C, 1.4 hours (0.8 to 8.0) at 38.0°C, and 4.5 hours (1.0 to 20.8) at 37.5°C (Fig. 3).

### Sensitivity Analyses

**Figure 4:**
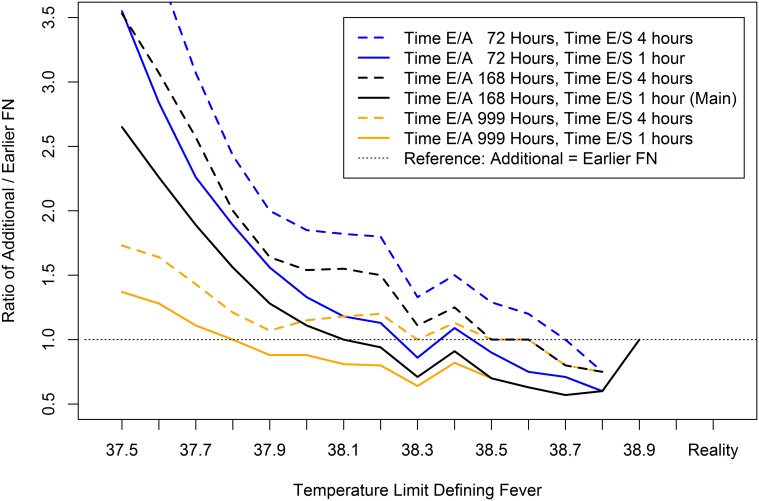
Ratio of additional/earlier FN diagnoses according to TLDF and definition of earlier FN diagnosis. Time E/A, time limit differentiating earlier versus additional FN. Time E/S, time limit differentiating earlier versus simultaneous FN.

In all 5 sensitivity analyses, the Limit_Low_ of 38.4°C found in the main analysis to result in the predefined increase of ≥15% in the FN rate (95% LCB of RR_Add/Std_ ≥0.15) remained unchanged. The ratio of additional versus early FN diagnoses at this temperature was 0.91 (10/11) in the main analysis, and ranged from 0.82 (9/11) to 1.50 (12/8) in the different sensitivity analyses (Table 2). The Limit_Low_ at and below which this ratio was always ≥1 was 38.1°C in the main analysis, and ranged from 37.8°C to 38.7°C in the different sensitivity analyses (Fig. 4).

### Adverse Events

An AE was reported in 14 (41%) of the 34 (32+2) FN episodes defined by Limit_Standard_, including bacteremia in 4 (12%), and a serious medical complication (SMC) in 1 (3%; diagnostic bronchoalveolar lavage in the intensive care unit for varizella zoster virus primoinfection with pneumonia; Table 2). Applying Limit_Low_ 38.0°C, an AE was reported in 8 of 18 earlier FN episodes versus 6 of 16 simultaneous FN episodes (44% versus 38%, p = 0.74). Overall, no significant differences were found for AE, bacteremia, and SMC between earlier and simultaneous FN episodes applying Limit_Low_ 38.5°C, 38.0°C, and 37.5°C, but the corresponding 95% CIs, and thus uncertainty, were very large.

### Clinical Course in Reality

The clinical course of FN in reality was analyzed in the full dataset, i.e., without discarding information on the 7 days preceding the 11 FN episodes diagnosed at Limit_Reality_<Limit_Standard_. Applying Limit_Low_ 38.0°C, 65 FN episodes would have been diagnosed in this full dataset, i.e., 31 (65–34) more FN episodes than applying Limit_Standard_ 39.0°C (Table 3).

**Table 3:** Clinical Course of FN Episodes Diagnosed in Reality, and Virtually at Different Temperature Limits Defining Fever.

	**FN diagnosed**	**FN virtually diagnosed using limit_Standard_ or limit_Low_**				
**FN episodes**	**in reality**	**39.0°C**	**38.5°C**	**38.4°C**	**38.0°C**	**37.5°C**
Total number of episodes^a^	43 (100%)	34 (100%)	49 (100%)	53 (100%)	65 (100%)	98 (100%)
* Episodes with adverse event*	*19 (44%)*	*14 (41%)*	*19 (39%)*	*19 (36%)*	*20 (31%)*	*21 (21%)*
Diagnosis of FN, intravenous antibiotics	43 (100%)	32 (94%)	40 (82%)	41 (77%)	43 (66%)	43 (44%)
* With adverse event*	*19 (44%)*	*14 (41%)*	*18 (37%)*	*18 (34%)*	*19 (29%)*	*19 (19%)*
* Without adverse event*	*24 (56%)*	*18 (53%)*	*22 (45%)*	*23 (43%)*	*24 (37%)*	*24 (24%)*
No diagnosis of FN, oral antibiotics	0	2 (6%)	3 (6%)	3 (6%)	3 (5%)	4 (4%)
* With adverse event*	*0*	*0*	*1 (2%)*	*1 (2%)*	*1 (2%)*	*2 (2%)*
* Without adverse event*	*0*	*2 (6%)*	*2 (4%)*	*2 (4%)*	*2 (3%)*	*2 (2%)*
No diagnosis of FN, no antibiotics	0	0	6 (12%)	9 (17%)	19 (29%)	51 (52%)
* With adverse event*	*0*	*0*	*0*	*0*	*0*	*0*
* Without adverse event*	*0*	*0*	*6 (12%)*	*9 (17%)*	*19 (29%)*	*51 (52%)*

FN, fever in neutropenia.

^a^Full dataset.

Because of poor general performance of the patients, an FN diagnosis implying empirical intravenous antimicrobial therapy had been made in 11 (43–32; 35%) of these 31 additional FN in reality. AE were reported in 5 of these 11 FN episodes diagnosed at temperature below Limit_Standard_: The first patient had a positive blood culture (*Moraxella catarrhalis*), herpes zoster, candidiasis of the skin, and a bronchoalveolar lavage (negative results) was performed in the pediatric intensive care unit. The second patient had an upper respiratory tract infection with picornavirus detected. The third patient had a positive blood culture (*Enterococcus faecium*). The fourth patient had a positive blood culture (*Fusobacterium* sp.). The fifth patient had a multifocal osteomyelitis, with *Campylobacter* sp. detected by polymerase chain reaction (Table 3).

Oral antibiotics had been given in 1 (3–2; 3%) of these 31 additional FN, in which an AE was reported (upper airway infection with beginning pneumonia). No antimicrobial therapy had been given in the remaining 19 (19–0; 61%) of these 31 additional FN, of which all had an uneventful clinical course without AE (Table 3).

## Discussion

The results of this study demonstrate the efficacy of a high versus low TLDF by significantly reducing the number of FN diagnoses, emergency calls, and CBCs performed for fever. Specifically, virtually lowering the TDLF from 39.0°C to 38.4°C led to a clinically relevant increase of FN diagnoses exceeding 15%. In reality, spontaneous defervescence without specific therapy and without AE was observed in most of these additional FN virtually diagnosed at lower TLDFs. Lowering Limit_Low_ to around 38.0°C [2, 3] a steady increase of emergency calls, emergency CBCs, and FN diagnoses was observed. This increase became steeper when Limit_Low_ was lowered further.

Reducing the number of FN diagnoses is an extended version of risk-adapted treatment restriction in diagnosed FN [7, 15, 19–21]. Less FN diagnoses imply less emergency hospitalizations, less empirical therapies with intravenous antibiotics, less costs, and supposedly a better quality of life for patients [9–11, 22, 23].

This study was not designed to assess the safety of a high TLDF, but safety was indirectly, and only roughly, estimated in three ways. First, FN episodes diagnosed in reality below Limit_Standard_ were recorded. Their number, a quarter of all FN, was non-negligible. These episodes reflect that the treating physician was free to diagnose FN at temperatures below Limit_Standard_ if clinically indicated, as suggested by current guidelines [2]. In nearly half of these FN episodes diagnosed below Limit_Standard_ an AE was reported. This predefined priority of clinical impression over the TLDF clearly increases the safety of the high Limit_Standard_ used. Second, the difference in FN diagnosis time applying Limit_Low_ versus Limit_Standard_, Time_Earlier_, was calculated as a potential surrogate marker of safety. Earlier diagnosis implies earlier start of empirical antibiotic therapy, which in turn may decrease morbidity and mortality in patients with bacterial infection [12]. Time_Earlier_ was non-negligible for many FN episodes in a large range of TDLFs (Fig. 3). Correspondingly, the number of earlier FN was as well non-negligible. It was notably higher than the number of additional FN for TLDFs ≥38.2°C. Sensitivity analyses showed that both these numbers and thus their ratio heavily depended on the time limits differentiating earlier versus simultaneous FN, and earlier versus additional FN (Table 2, Fig. 4). Third, AE were compared between earlier and simultaneous FN episodes applying different TLDFs. No significant differences were found for AE in general, for bacteremia, and for SMC, but these comparisons were clearly underpowered. Taking these three findings together, a TLDF of 39.0°C as used in Bern might prove unsafe in larger studies. However, the fact that the treating physician was free to diagnose FN at lower temperatures if clinically indicated, lessens this problem: In a quarter of FN episodes, the diagnosis was made without—or probably before—Limit_Standard_ was reached for clinical reasons. The fact that relevant AE were detected in nearly half (5 of 11) of these episodes underlines the importance that TLDFs, be they low or high, must not be used as absolute limits neglecting other clinical findings.

To our knowledge this is the first prospective, though purely observational, study of the effect of lowering the TLDF on the rate of FN in pediatric or adult oncology. Its results clearly contradict the counterintuitive finding of no association between TLDF and FN rate in the only retrospective study in pediatric oncology [6]. Methodological weaknesses seem to have led to false negative findings there.

The findings of this study are based on large numbers of temperature measurements and CBCs prospectively recorded. This study relied on the high TLDF of 39.0°C used in Bern. A reverse design, i.e., virtually assessing the impact of higher TLDFs in centers using low or medium TLDFs, is made impossible by the routine application of antipyretics after FN diagnosis. This implied a single-center study design, with its potential inherent limitations on generalizability of results. In silicon simulation allowed for a non-interventional study. Together with a dedicated PI and research nurse, both known to all patients and parents from clinical routine, these aspects have led to the near-perfect accrual rate which sharply contrasts recent reports on supportive care studies in pediatric oncology [24]. In the outpatient setting, the study relied on reporting of temperature measurements by parents. Incomplete reporting may have led to underestimated rates of both additional and delayed FN diagnosis applying Limit_Low_, This might be prevented by using temperature measurement devices that automatically store time-stamped results.

In conclusion, this study showed that a high TLDF of 39.0°C is efficacious via reducing FN diagnoses. Lowering the TLDF to 38.4°C, and further to 38.0°C [2, 3] led to a relevant number of additional FN diagnoses, implying overtreatment in the majority of them because spontaneous defervescence was observed in reality. However, it would as well have led to earlier diagnosis, and thus earlier start of therapy, in the majority of episodes. The question if a high TLDF is safe remains open. Before clinical application of this high TLDF in other centers, however, the question of safety must be reliably answered. Even a slight decrease in safety, with its implications on morbidity and mortality, would need to be cautiously weighed against a relevant decrease in hospitalizations and antimicrobial therapy. Based on the results of this study, a large randomized controlled multicenter trial adequately powered to answer this safety question is currently under development.
